# Chemistry publication – making the revolution

**DOI:** 10.1186/1758-2946-1-2

**Published:** 2009-03-17

**Authors:** Steven M Bachrach

**Affiliations:** 1 Department of Chemistry, Trinity University, One Trinity Place, San Antonio, Texas 78209 USA

## Abstract

The advent of the Internet has been the impetus for the Open Access movement, a movement focused on expanding access to information principally by reducing the costs of journals. I argue here that the Open Access movement has had little impact on the chemistry community and has taken our attention away from the real opportunity to revolutionize scientific communication. I propose a plan that both reduces the total cost of publishing chemistry and enriches the literature through incorporation of Open Data. By publishing lots of data, available for ready re-use by all scientists, we can radically change the way science is communicated and ultimately performed.

## Commentary

How are journals failing us? It seems reasonable that before we as a chemistry community attempt to reconstruct the journal, we should assess what is wrong with the current incarnation and then design a corrective solution. I will limit the scope of this polemic to the chemistry community, the community in which I am an active member.

Perhaps the most commonly stated failing of scientific journals is their astounding cost. Journals are very expensive, often prohibitively so. As a measure of this, consider the rate of inflation across all journals provided by the Association of Research Libraries [1]. From1986 to 2006, the increase in the average journal subscription price is 180%. It should be noted that the unit journal cost has in fact decreased from its peak in 2000, an issue I will address later on. Even with this recent price decline, library expenditures have systematically increased every year from 1986 through 2007, an overall increase of 340% [2].

How have libraries responded to these fantastic increases in costs? Generally, the effect has been that each institution subscribes to ever fewer journals and purchases ever fewer monographs. The net result is that we scientists have direct access to a smaller portion of the overall scientific output. And this is what is meant by the "journals crisis" – a limiting of the ability to read the scientific literature due solely to lack of funds. In effect, the sole purpose of the journal – a means for disseminating scientific knowledge to all – is disrupted. Instead of reaching all chemists, our literature reaches only those that can afford to pay for access.

This problem is acute in our discipline. Table 1 lists the average serial cost per discipline [3]. This table lists the five most expensive disciplines in the science and technology arena, and chemistry leads the pack.

**Table 1:** Average cost (2008) per journal for specific scientific discipline[3].

Discipline	Average journal cost
Chemistry	$3,490
Physics	$3,103
Engineering	$1,919
Biology	$1,810
Technology	$1,776

Perhaps more disturbing than the fact that our journals are the most expensive of all science and technology disciplines is that the rate of inflation of chemistry journals subscription fees is staggering [3]. The annual inflation rate for chemistry journals has been 6% or greater (Table 2), leading to an overall increase of 35%, a pace far outstripping the consumer price index (CPI) of the United States for the same period.

**Table 2:** Rate of inflation of chemistry titles [3].

Year	Avg. cost/title	% annual increase	CPI^*a*^
2004	2,582		2.7
2005	2,748	6	3.4
2006	2,965	8	3.2
2007	3,187	7	2.8
2008	3,490	9	

^*a*^Consumer price index, a measure of inflation in the United States, see United States Department of Labor, Bureau of Labor Statistics, http://www.bls.gov/cpi/.

### Enter Open Access

The motivation behind the Open Access [4] (OA) movement has been to increase the availability of the literature. The principle impediment to reading journals, particularly scientific journals, is their high subscription costs. The OA community has focused their efforts towards reducing the expense for the reader. While there are many definitions of Open Access, and many different flavors of how Open Access journals are implemented, I will use what is likely the most common interpretation of Open Access for this commentary, and that is articles that are available at no cost to the reader. Clearly, this idea solves the cost issue for readers who can get to the article for no fee at all. It does not solve the global cost issue, as addressed below.

The downward trend in journal unit cost seen in the ARL data [1] in the early part of the current decade is likely due to the Open Access movement. Undoubtedly the acceptance and growth of the Public Library of Science [5] and the BioMed Central [6] journals, amongst others, have reduced subscription costs across the science and technology field.

But what has been the impact of Open Access within chemistry? Well, the rate of inflation of chemistry titles (Table 2) appears to be unaffected; publishers continue to raise prices at an exorbitant rate. The *Directory of Open Access Journals *[7] lists 75 chemistry journals. Perhaps the two most notable OA chemistry journals are *Chemistry Central Journal *[8] and *Beilstein Journal of Chemistry *[9]. In the two years of its operation, *Chemistry Central Journal *has published about 50 articles, and in its four year history, *Beilstein Journal of Chemistry *has published about 125 articles. The American Chemical Society introduced in 2006 *Author Choice *[10], its OA option where authors can pay to make their articles available to readers at no cost; some 70 articles are available through this program. When weighted against the total number of articles published over the past four years (see below), very few chemists have opted to publish in OA journals, and the net effect is that OA has had a negligible impact on chemistry.

Nonetheless, Open Access had garnered a great deal of attention and many devoted followers. OA proponents strongly believe that the interest of science is best served by the widest distribution of scientific information, and that no barriers should be placed on any reader gaining access to any paper. This philosophy is best embodied by Stewart Brand's mantra: "Information wants to be free" [11]. Also embodied in the OA philosophy is a concept of "fairness"; scientific information should not be available only to the wealthy, but to all scientists, and even the general public should have full access. Since much research is funded by government agencies, so society as a whole has paid for this research, OA proponents argue that the results of this publicly-funded research should be universally available at no additional cost [12]. Others argue that third-world countries and other impoverished users should not be prohibited from gaining access to important discoveries solely because they lack sufficient funds to pay for access.

It should be recognized that *any publication means requires funds for operation*. Journals will require editors, copy-editors, a mechanism for peer review, internet connectivity and servers, and printing and shipping if hardcopy is produced. It is unclear how any OA model leads to a reduction in net costs relative to the traditional journal subscription model, other than perhaps an altruistic notion that removes or reduces the profit margin. Most OA journals operate on an author-pays model, and so it appears that the cost of publication simply is shifted from the reader to the author. Where are the cost savings?

A further concern I have is whether the author-pays model is truly "fair". Under the current subscription-based system, all readers pay to gain access. Many publishers have a tiered pricing system whereby individuals pay less than a library, and, often, corporate libraries pay more than academic libraries.

In the author-pays model, who are the winners and who are the losers? Small universities and colleges are likely to do well by the author-pays model; their faculties tend to publish at low rates. For example, my own department averages about 15 publications per year, and with a publication fee of $2000 per article, our total cost would be $30,000 – much less than the approximately $100,000 we currently pay for chemical information.

On the other hand, large research universities will face the lion's share of the publication cost. Perhaps some of this burden could be alleviated by changes in funding agency policies, which may allocate additional funds to cover these costs. (Currently, some agencies will allow for publication charges, but in the past these types of expenses have been much smaller than the price OA publication.)

How will the corporate-world fare under the author-pays model? Under the current subscription model, Michael Mabe (Elsevier) estimated that corporate subscription fees account for about 17% of revenue [13]. He estimated that corporate publications amount to only 5% of all publications; this would translate into a tremendous cost savings for industry under the author-pays model if (a) the fee assessed all authors is identical and (b) if corporate scientists continue to publish at the same rate. Of course, if companies will have to pay to publish, it is not clear what incentive remains for corporations to publish – public relations may only go so far in the mind of a bottom-line oriented manager.

I have carried out a SciFinder [14] search to try to assess the amount of corporate-based publication in chemistry. I identified all articles whose "company/organization" field contained any of the words "college", "university" or "institution". All those remaining articles I assume are of corporate origin, though this is likely to overestimate the non-academic origins. Table 3 lists the total number of articles and the percent that originate from non-academic sources for the period 2003–2007.

**Table 3:** Non-academic sources of published chemistry articles.

Journal	Total articles	% non-academic
*Journal of the American Chemical Society*	16794	8.1
*Angewandte Chemie*	8928	19.5
*European chemistry journals*^*a*^	10901	13.2
*Journal of Organic Chemistry*	7705	8.0
*Tetrahedron *and *Tetrahedron Letters*	17171	16.2
*Journal of Physical Chemistry *and *Journal of Chemical Physics*	34799	10.4
*Journal of Medicinal Chemistry*	6532	24.5
*Journals with "Drug" in their titles*^*b*^	17412	37.7

^*a*^*Chemistry* a *European Journal*, *European Journal of Organic Chemistry European Journal of Inorganic Chemistry* and *European Journal of Medicinal Chemistry*. ^*b*^149 titles, the top five are *Nature Reviews – Drug Discovery*, *Drug Metabolism & Discovery*, *Drug Development & Industrial Pharmacy*, *Advanced Drug Delivery Reviews*, *Current Drug Targets*.

The data in Table 3 support the contention that most of the chemistry literature originates in academe. This is especially true for the more general literature (*Journal of the American Chemical Society, Angewandte Chemie*) and for most subdisciplines. The exception is in the area of drug design, as might be expected. One has to wonder, however, if companies will continue to publish in the open literature if they are required to pay for each publication. Might not corporate science become solely published in the patent literature, if not just secreted away within their own walls forever? Industry might just be the major beneficiary of an author-pays model!

**Figure 1:**
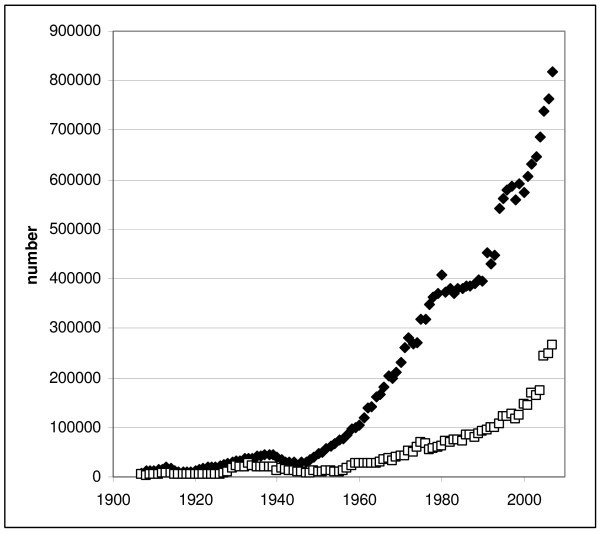
**Number of articles (diamonds) and patents (open boxes) abstracted annually by *Chemical Abstracts ***[16].

I have argued previously that the real cause of the journals crisis lies with the scientists themselves [15]. We simply publish way too much. Figure 1 displays the growth curve for the number of articles and patents abstracted by *Chemical Abstracts *for the past century [16]. It is notable that we chemists exceeded the annual one million publications mark in 2006, with no end to this growth curve in sight.

No one can keep up with a literature like this. It is time for the scientific community to rethink the role of publication. Should every little idea, every minor work receive the same treatment as the great discovery? By this I mean, regardless of the quality of the science, the process of publication is identical: an author writes the article in the appropriate fashion, sends if off to a journal for external peer review, once revised the manuscript is copy-edited, typeset and then put onto paper and onto a computer and disseminated around the world. Most articles barely get cited – many never get cited; they are simply maintained within this ever-growing collective of scientific work, with the chaff growing at a rate exceeding the production of the wheat.

What is it about journals that we hold so dear? We want accurate scientific information to be available to anyone who needs it at any time or place. Appendix 1 presents the needs of the science authors in terms of the publication mechanism. I have broken these down into the groups of persons responsible for carrying out these aims.

I'll argue that most of these aims are being met under the current publication model. Publishers have reduced the average time from acceptance to publication of the average paper and have accepted the mantle of archiving journals, often in collaboration with librarians [17]. As argued above, there is some doubt as to whether articles are widely disseminated due to cost, but there can be no doubt that the rise of the internet and electronic journals means that science is available in principle anywhere with a wi-fi or broadband connection.

My main arguments rising from this list are two-fold. First, I do not think that editors and editorial boards have done a good job in managing the burgeoning literature. Individuals have been too quick to join editorials boards of new journals without stopping to think whether the market need is not already being fulfilled. For example, just what community is being denied access to their science that requires the introduction in 2009 of *Nature Chemistry *[18]? When new sub-disciplines emerge and reach a level of maturity, a new journal might be warranted, but great care should be taken, not just the thought of yet another profit center.

Furthermore, extant journals are growing fatter and fatter. Is this healthy? Are there really a million articles worth publishing? Are we placing our limited resources in appropriate places? Is the time spent on peer-reviewing all of those articles really worth it, when many published articles are destined to never be cited? Is subscribing to all these journals really necessary?

One could imagine removing some of the strain of peer-review by adopting some Web 2.0 technologies [19]. Peer review could be taken on by the community as a whole, using forums and wikis and blogs. ArXiv [20], the preprint server of the high energy physics community and other disciplines, creates a forum attached to each article and the community can (and does) comment. A number of individuals, myself included [21], regularly blog on the literature [22]. The GreaseMonkey [23] plugin to Firefox [24] along with the PostGenomic [25] toolset and the Chemical Blogspace [22] web site allow for pop-ups to appear on journal table of contents pages indicating the presence of blog commentary. This concept is extended to linking to other web sites and databases through the use of userscripts [26]. Tagging of articles using Technorati-like tags [27], and rating systems can be used to steer others to useful work and to avoid other articles.

Unfortunately, the scientific publishers have been slow to adopt Web 2.0 tools. According to the Association of Learned and Professional Society Publishers Survey [28] "Just 20 percent [of current journals] enable collaborative tagging, and less than 15 percent have implemented things like forums, blogs, or podcasts for their journals." More can certainly be done here!

My primary concern with scientific publishing, one that I and others have been highlighting for a decade [29-32], is that we are missing the revolution that the Internet and electronic publication offers us. To me, this is the greatest current failure of scientific journals. Much of science today is lost in the publication process. Information is omitted due to space limitations. Information is published in ways that make it difficult, if not impossible to re-use. Both of these issues can be rectified today with the revolution in electronic publication. *Journals can be information-rich, containing raw data and data preformatted for direct re-use by the reader. Since disk space is cheap, there should be no limits on article size. All data that the authors had at hand for reaching their conclusions should be made available to the reader*.

Let me provide two simple examples of how the current system corrupts and stymies information dissemination.

• NMR spectra are not typically reported as spectra, but rather as a list of chemical shifts (and multiplicity for ^1^H spectra). Even viewing the actual drawn spectra would be preferable, allowing one to see minor signals and impurities that might have been overlooked. Preferable still would be to have the spectral data file available for direct import into one's favorite spectral viewing program, allowing the reader to manipulate the spectrum, overlay it with one's own sample, and work fully with all the data.

• Molecular structures are often made available as a poorly reproduced image and perhaps a table of selected distances and angles. Why not deliver to the reader a coordinate file that can be directly piped into a molecular visualization program for manipulation to the reader's heart's content?

What will drive future scientific development is the rapid and complete dissemination of data in usable, customizable formats with no data loss. We need to publish *datuments *instead of *documents*. The concept of *datument*, introduced by Murray-Rust and Rzepa, suggests that scientists should be "transmitting and preserving the complete content of a piece of scientific work" when they publish [33]. Readers will be able to get their hands on all the data available to the authors. Errors will be more easily traced and corrected. Access to the full data sets will inspire new insights and new experiments.

It is my contention that the Open Access movement, by concentrating on journal costs, has diverted our attention and focus away from the potential publication revolution that the Internet can empower. Fortunately, I believe it is not too late and we can still remake the journal in a way that can revolutionize how we do science.

I offer a two-tier publication model that attempts to address both the ever-increasing cost spiral and to bring new technologies to bear on scientific communication. I note in passing that the Open Notebook Science [34] initiative is an even more disruptive [35] publication model than the one I propose here; Open Notebook Science involves publishing science *as it is performed *and made available to all for comments and collaboration. While there is much of interest in this model, I believe that it is too radical a change for the chemistry community to adopt in the near term.

### Tier 1 publishing

We need to reduce the number of full-service, full-feature journals and the number of articles they publish. I believe that each of us has in their minds a hierarchy of journals – those in which we publish our best works and which we read religiously, those we peruse on occasion and those that we consider rubbish. Each of us should support only those top-tier outposts of science. We should publish in, peer-review, serve on editorial boards, and ask our libraries to purchase just these journals. For all others we should decline our services and our moneys. We should end up with perhaps 20% of the total number of current journals.

I offer this small anecdote to support this notion of a real hierarchy of importance within the journals. I recently authored the monograph *Computational Organic Chemistry *[36], which surveys the achievements in the field over the past 40 years. In it, I cite 935 articles. Table 4 lists the top ten sources of these citations. These top ten journals account for 73.3% of the citations, clearly indicating the dominance of the major journals. Now the citations within a monograph surveying a different subject will give rise to a different set of journals, but it is likely that the major journals (*JACS, Angewandte Chemie, Nature, Science*, etc) will dominate.

**Table 4:** Top ten cited journals in the book *Computational Organic Chemistry *[36].

Rank	Journal	# citations
1	*Journal of the American Chemical Society*	351
2	*Journal of Organic Chemistry*	65
3	*Journal of Physical Chemistry A*	64
4	*Journal of Chemical Physics*	58
5	*Angewandte Chemie*	38
6	*Chemical Physics Letters*	25
7	*Organic Letters*	24
8	*Tetrahedron Letters*	22
8	*Journal of Physical Chemistry*	22
10	*Journal of Computational Chemistry*	17

This is not to say that we can completely do without all of the rest of the literature. Clearly, the other quarter of the citations in the book are important too! This brings me to the second tier.

### Tier 2 publishing

The remaining say 80% of the scientific literature should be "published" within institutional repositories (IR) [37,38]. I place quotations around the word "publish" to indicate that this effort is quite different in kind from what is carried out in Tier 1; Tier 1 publishing includes all of the components we commonly associate with the publishing endeavor (Appendix 1): full-time staff of editors and copy-editors, peer review of articles, typesetting, widespread dissemination and archiving. Institutional repositories will provide just the role of dissemination and archiving. Authors will write and typeset the articles themselves, and then deposit in the IR. Peer review will occur by way of the community interacting through Web 2.0 tools, such as forums, wikis, tagging and blogs, which originate within the IR or are linked into it.

Each institution will establish their own IR or combine with a number of institutions to create a consortial IR. Each institution will supply the funds to maintain the IR: a server, internet connection, software, and maintenance. IR will operate with a standard protocol that allows interlinking and cross-searching through meta-tags. Initiatives in this effort are underway, notably the Open Archives Initiative Protocol for Metadata Harvesting (OAI-PMH) [39].

Cost savings comes about in this model because only about 20% of the literature will end up in the full-featured journals, the ones that will continue to require a staff and a significant operating budget. These journals can be subscription-based or author-pays or even some third method – the important point is that there will be far fewer journals. The operation of the institutional repositories will require some funds, but since these repositories will offer only a very limited set of publication functions, they can be operated at a far lower cost than currently appropriated, leading to the ultimate cost savings.

But my main advocacy within this piece is for the development of tools that will lead to an enriched publication that offers new avenues for real scientific discourse and development, the revolution that the Internet enables. The key is to publish Open Data [32,40] – publication of lots and lots of data freely available to all with no restrictions on re-use, available in the format that allows the reader ready and direct access for complete reuse. This can be accomplished by actions from all interested parties:

• **Authors **should include more data within the supporting materials of journals. While most journals provide supporting (or supplementary) materials, they do so in only limited formats like pdf. Authors should include data – coordinate files, spectra, kinetics output, etc – in whatever format is most suitable that the journal allows, but each author should advocate for the ability to deposit more appropriate formats. Authors should ask for more tools for preparing supplementary materials in appropriate formats for deposition.

• **Reviewers **should flag those manuscripts that lack supporting data and insist that authors include it as part of the publication.

• **Editors **hold a great deal of power in this battle. We see this in the area of x-ray crystallography, where editors many years ago required that structures be deposited in the Cambridge Structural Database (CSD) [41] as part of the submission process. This decision has the happy outcome that a virtually complete database of x-ray structures is available; unfortunately the CSD is available only for a fee. Many journals have started to require that the x-ray structures be deposited as supporting materials in addition to being deposited in the CSD. Peter Murray-Rust's group has demonstrated in the CrystalEye project [42] how these open data files can be mined, collated, and made searchable. Editors should broaden the requirements for submission of articles to mandatorily including all data used within the paper. Editors should further use their influence to insist that the publishers make these data available under the Open Data model – unlimited access and re-use without any payment or restriction.

• **Publishers **should welcome the additional supporting materials and make then available under the Open Data principle. Furthermore, publishers should encourage the mining of these supporting materials, like the CrystalEye [42] and ChemSpider [43] projects. This mining will only serve to drive more traffic to the journal articles, since scientists will find more pertinent information through these mining tools and then wish to get to the source!

• **Software Developers **must play a key role in this system. While the editors and publishers are pivotal to its success – authors won't likely submit all of their data unless forced to do so – the submission of data (and lots of it) will only occur if the submission process is painless. Making this process painless requires development of software tools. These tools should create formatted data files with as little user-input as possible; the ideal situation is one where the user doesn't even know that a formatted file has been produced! In addition, software developers, working with the publishers and database manufacturers should create and establish appropriate data formats for widespread use. Examples of data formats of this type include the IUPAC International Chemical Identifier (InChI) [44], Joint Committee on Atomic and Molecular Physical Data Spectroscopic Data Standards (JCAMP-DX) [45], and Chemical Markup Language (CML) [46-50].

Let me describe some examples of "necessary tools". These tools need to operate transparently in the sense that they produce the properly formatted and meta-tagged files without any author intervention.

• An author draws a two-dimensional chemical structure and embeds this picture into her document. Along with the "dead" image comes a connection table and an InChI, all formatted and ready for indexing and reuse.

• An author uses his favorite program for spectral manipulation, creating an image of the spectrum suitable for publication. This image is pasted into the document. Along with the "dead' image comes the JCAMP data, an InChI, and meta-tags describing, for example, the type of spectrometer and experimental conditions.

• An author uses her favorite program to visualize the three-dimensional structure of a molecule. She selects an orientation that suits her needs and embeds this image into her document. Along with this "dead" image comes the 3-D coordinates, the InChI, and meta-tags indicating how the structure was obtained – for example, as a result of a computation at a certain level.

The last piece of the puzzle is the creation of a utility within a word-processing program to extract out these data files and meta-tags and automatically generate the supporting materials. Within a model like this, authors would continue to work and draft articles essentially as they have been for years. The only change would be hitting the "publish" button that would generate the appropriate supporting materials, and then the authors would submit both the article and this supporting material. By creating a system of intercommunicating tools, a publishing work-flow utility can be put into place that imposes few requirements or behavioral changes on scientists. In this manner, I believe we can revolutionize how we publish our science.

There are two fledgling experiments that attempt to put into place some of these enhanced-publication/datument ideas: Project Prospect [51] from the Royal Chemical Society and ChemMantis from the ChemSpider [43] group.

We need to encourage these projects and the development of more tools. We need to encourage our colleagues to adopt a new model for publication. We can revolutionize how we perform our science. This is the real hope of the internet for scientists.

## Competing interests

The author was the editor of a for-profit journal (*Internet Journal of Chemistry*) that advocated many of the ideas expressed in this paper. That journal is now defunct. The author is on the Advisory Board of the *Chemistry Central Journal*. The author's wife is employed by Symyx Technologies.

## Appendix 1

### Goals of science publication

Wide distribution

Rapid communication

Archived for future access

Publisher

Content is meaningful

Peer review

Content is managed and cohesive

Editors and editorial boards

Content is written in grammatical correct style

Content is presented in a consistent fashion

Copy-editor
